# Comparative analysis of gut microbial composition and potential functions in captive forest and alpine musk deer

**DOI:** 10.1007/s00253-022-11775-8

**Published:** 2022-01-17

**Authors:** Feng Jiang, Pengfei Song, Haijing Wang, Jingjie Zhang, Daoxin Liu, Zhenyuan Cai, Hongmei Gao, Xiangwen Chi, Tongzuo Zhang

**Affiliations:** 1 Key Laboratory of Adaptation and Evolution of Plateau Biota, Northwest Institute of Plateau Biology, Chinese Academy of Sciences, Xining, 810001 Qinghai China; 2 University of Chinese Academy of Sciences, Beijing, 100049 China; 3 Qinghai Provincial Key Laboratory of Animal Ecological Genomics, Xining, 810001 Qinghai China; 4 Qinghai University, Xining, 810016 Qinghai China; 5 Present Address: Northwest Institute of Plateau Biology, Chinese Academy of Sciences, Chengxi District, 23 Xinning Rd, XiningQinghai Province, 810001 China

**Keywords:** Musk deer, Gut microbiota, Dominant bacteria, Metabolic functions, 16S rRNA gene sequencing, Disease-related functions

## Abstract

**Abstract:**

Gut microbiota forms a unique microecosystem and performs various irreplaceable metabolic functions for ruminants. The gut microbiota is important for host health and provides new insight into endangered species conservation. Forest musk deer (FMD) and alpine musk deer (AMD) are typical small ruminants, globally endangered due to excessive hunting and habitat loss. Although nearly 60 years of captive musk deer breeding has reduced the hunting pressure in the wild, fatal gastrointestinal diseases restrict the growth of captive populations. In this study, 16S rRNA high-throughput sequencing revealed the differences in gut microbiota between FMD and AMD based on 166 fecal samples. The alpha diversity was higher in FMD than in AMD, probably helping FMD adapt to different and wider habitats. The ß-diversity was higher between adult FMD and AMD than juveniles and in winter than late spring. The phylum *Firmicutes* and the genera *Christensenellaceae R7 group*, *Ruminococcus*, *Prevotellaceae UCG-004*, and *Monoglobus* were significantly higher in abundance in FMD than in AMD. However, the phylum *Bacteroidetes* and genera *Bacteroides*, *UCG-005*, *Rikenellaceae RC9 gut group*, and *Alistipes* were significantly higher in AMD than FMD. The expression of metabolic functions was higher in AMD than in FMD, a beneficial pattern for AMD to maintain higher energy and substance metabolism. Captive AMD may be at higher risk of intestinal diseases than FMD, with higher relative abundances of most opportunistic pathogens and the expression of disease-related functions. These results provide valuable data for breeding healthy captive musk deer and assessing their adaptability in the wild.

**Key points:**

*• Alpha diversity of gut microbiota was higher in FMD than that in AMD*

*• Expression of metabolic and disease-related functions was higher in AMD than in FMD*

## Introduction

Gut microbiota and host evolve together, forming a complex microecosystem within the gastrointestinal tract of animals, which functions in material metabolism, nutrient absorption, immune regulation, resistance to pathogen invasion, and other host physiological processes (Nicholson et al. 2012). Gut microbiota is a complex and dynamically balanced ecological network, jointly maintaining the gut environment homeostasis and the health of the host (Hua et al. 2020). Imbalanced gut microbiota causes partial host dysfunction and significant changes in the host immune response, seriously affecting host health and growth (Gagniere et al. 2016). Recent studies have demonstrated that gut microbial structure abnormalities are associated with mental disorders, intestinal, metabolic, and other diseases (Dinleyici et al. 2018; Qin et al. 2012; Fung et al. 2017; Valles-Colomer et al. 2019). Moreover, various intrinsic and extrinsic factors such as host genetics (Fan et al. 2021), genetic background (Korach-Rechtman et al. 2019), diet (Wang et al. 2019), age (O’Toole et al. 2015; Guo et al. 2020a), seasonal change (Peddada 2017), and habitat environment (Barelli et al. 2020; Xiong et al. 2021) greatly affect gut microbial community structure.

In herbivorous mammals, gut microbiota secretes exogenous cellulases and hemicellulases that convert plant biomass into absorbable nutrients and energy (Naas et al. 2018). Ruminants possess a unique gastrointestinal microbiome and a specialized, compartmentalized digestive system consisting of the rumen, reticulum, omasum, and abomasum. The rumen is involved in microorganism fermentation, food digestion, material, and energy metabolism (Matthews et al. 2019; Prajapati et al. 2016). Food first enters the rumen of ruminants, where rumen microorganisms perform catabolism, and is further transferred into the reticulum for finer catabolism. Next, the omasum and abomasum degrade and convert the food into small molecules to provide energy and nutrients to the host (Enjalbert et al. 2017). The ruminant gut microbiota contains probiotics, opportunistic pathogens, and pathogens. The genera *Bifidobacterium*, *Lactobacillus*, and other beneficial anaerobes dominate the ruminant gastrointestinal tract (Xu et al. 2018). These genera are mutual symbionts with the host, and their metabolites inhibit the propagation of opportunistic pathogens and hinder gut colonization by opportunistic bacteria (Lepczynska and Dzika 2019; Zhao and Qing 2021). The opportunistic pathogens are mainly facultative, non-dominant aerobic bacteria of the intestine (Sassone-Corsi et al. 2016). Under conditions such as compromised host resistance or an imbalanced gut microbiota, these opportunistic bacteria rapidly multiply and cause disease in the host.

For musk deers, gastrointestinal diseases caused by opportunistic bacteria are the main factors limiting the expansion of musk deer artificial breeding (Zhao et al. 2011; Fan et al. 2018; Zhou et al. 2019). The forest musk deer (FMD, *Moschus berezovskii*) and alpine musk deer (AMD, *Moschus chrysogaster*) are two types of solitary small and threatened ruminants that inhabit forests and mountains of central and southwestern China. China harbors the most diverse musk deer resource, quantity, and yield (Sun et al. 2018). The musk secreted by the ventral gland of the male musk deer is traditional Chinese medicine and a highly priced natural fragrance with limited supply. The limited musk supply has caused excessive hunting and habitat fragmentation of the wild musk deer, whose population has decreased dramatically from approximately 3 million in the 1950s to 31,800 in 2009 (Wu and Wang 2006; National Forestry and Grassland Administration 2009).

Both FMD and AMD are listed as endangered (EN) by the IUCN Red List and critically endangered (CR) by the Red List of Vertebrates in China (Wang and Harris 2015; Harris 2016; Jiang et al. 2016). In the late 1950s, China performed artificial FMD and AMD breeding, generating the largest captive population of musk deer species. This breeding relieved the resource pressure on wild populations and provided, to an extent, the traditional and natural musk resources (Huang et al. 2013; Fan et al. 2019). Artificial breeding of musk deer can also reintroduce provenance. For example, in 2017, 13 artificially bred FMD were released, for the first time, into the wild in Shaanxi Province, China (National Forestry and Grassland Administration 2017). The release was an important step in conserving endangered species, recovering, and expanding wild populations. However, captive musk deer are more susceptible to dysbiosis-caused intestinal diseases with higher incidence and mortality rates than wild musk (Li et al. 2018). Therefore, studying captive FMD and AMD gut microbiota is beneficial for evaluating their current artificial rearing conditions and understanding the appropriate capacity of gut microbial changes for future musk breeding. However, the differences in gut microbial composition and function between different ages of FMD and AMD in different seasons are still lacking.

In this study, 16S rRNA gene sequencing estimated FMD and AMD gut microbiota composition and diversity under different taxonomic levels. The aim was to explore (i) the difference between gut microbiota diversity between captive FMD and AMD, (ii) the difference between dominant gut bacteria and opportunistic pathogens in and between the two species, and (iii) the differences of potential metabolic and disease-related functions between the two species. This study comprehensively and systematically investigated the differences in gut microbial composition and potential function between FMD and AMD in different seasons and ages, providing a scientific basis for effective health management of captive musk deer.

## Materials and methods

### Sampled materials

**Fig. 1:**
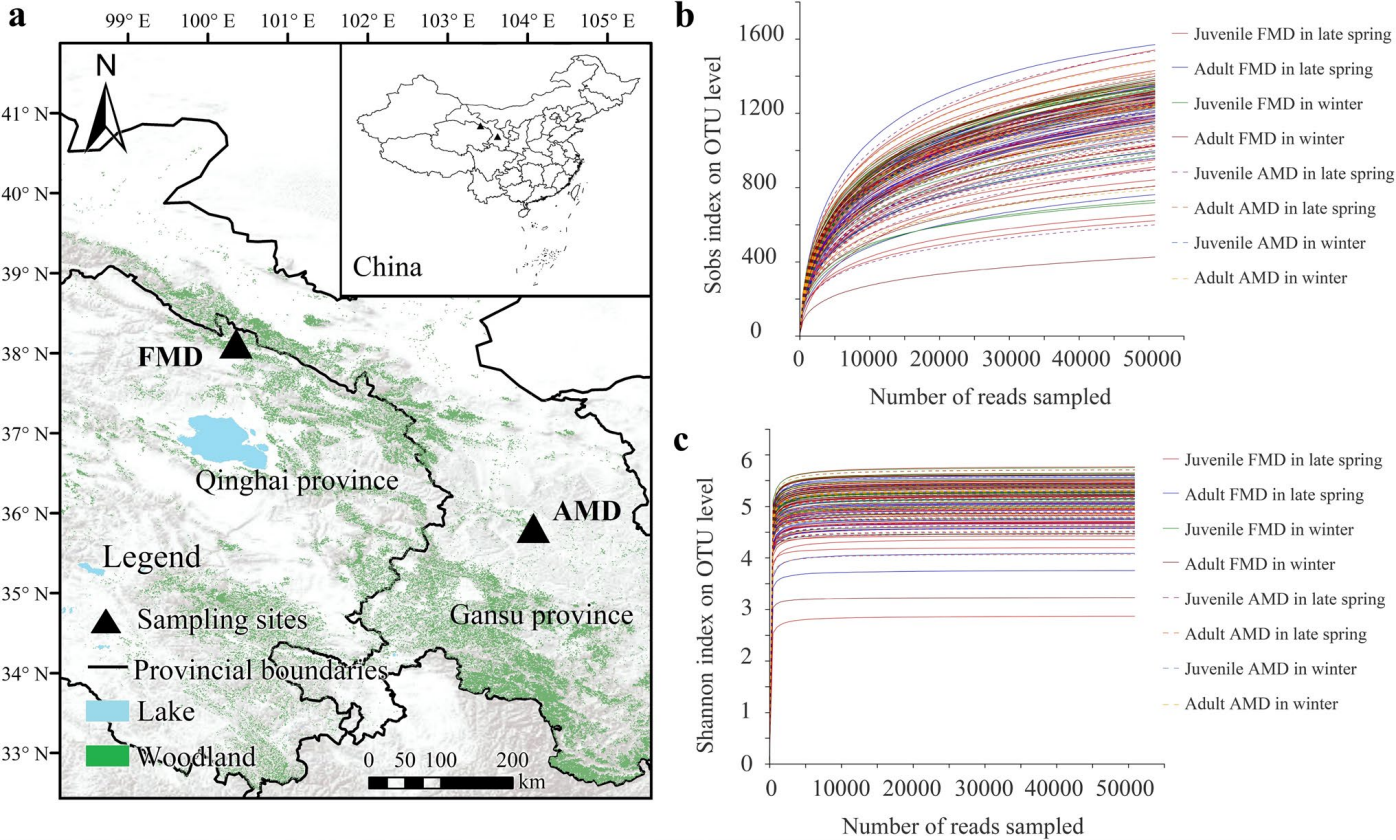
Diagram of sample collection of musk deer (**a**). Rarefaction curves of the 16S rRNA gene reads based on OTUs with *S*_obs_ index (**b**) and Shannon index (**c**)

A total of 107 fresh feces samples from captive FMD (57 samples in late spring and 50 samples in winter) and 59 samples from captive AMD (35 samples in late spring and 24 samples in winter) were collected by the noninvasive sampling method in this study. The FMD farm is located in the remote gully of A’rou Township, Qilian County, Qinghai Province (100°21′ E, 38°7′ N) (Fig. 1a), with an altitude of 3,002 m. The annual mean temperature and annual precipitation are − 0.1 °C and 403 mm, respectively. The AMD farm is located in the Xinglong Mountain National Nature Reserve in Yuzhong County, Gansu Province (104°4′ E, 35°49′ N), with an altitude of 2,171 m. The annual mean temperature and annual precipitation are 5.4 °C and 406 mm, respectively.

Before sampling, the individual enclosures of FMD and AMD were cleaned, and the individuals were kept in separate enclosures so that the fresh fecal samples could be collected from each individual the following morning. During the sampling process, fecal samples were collected shortly after a musk deer was observed defecating with sterile disposable polyethylene gloves and put into sterile bags. After labeling, all samples were temporarily stored in the vehicle-mounted refrigerator (− 20 °C) and later transferred into the − 80 °C ultra-low temperature refrigerator in the laboratory for later DNA extraction.

### DNA extraction and 16S rRNA gene sequencing

After pretreatment of fecal samples, total bacterial DNA was extracted using an E.Z.N.A.® soil DNA kit (Omega Bio-tek, Norcross, GA, USA) according to the manufacturer’s instructions, and subsequently stored at − 20 °C for further analysis. The quality of DNA extraction was determined by 1% agarose gel electrophoresis, and the concentration and purity of DNA in each sample were determined by NanoDrop2000 instrument (Thermo Fisher Scientific, Waltham, MA, USA).

The extracted total bacterial DNA was used as the template and universal bacteria primers 515F (5’-GTGCCAGCMGCCGCGG-3’) and 907R (5’-CCGTCAATTCMTTTRAGTTT-3’) were subjected to amplify the V4–V5 region of the bacterial 16S rRNA genes. PCR reactions for each sample were carried out in triplicate 20-μL reactions with 4 μL *TransStart* FastPfu buffer (5 ×), 2 μL dNTP mix (2.5 mM), 0.8 μL of each primer (5 μM), 0.4 μL *TransStart* FastPfu DNA polymerase, 10 ng sample DNA, and certified DNA-free PCR water up to 20 μL. PCR amplifications were performed on an ABI GeneAmp 9700 PCR system (Applied Biosystems, Foster City, CA, USA) according to the following procedures: 98 °C for 3 min (initial denaturing), 27 cycles of 95 °C for 30 s (denaturing), 55 °C for 30 s (annealing), 72 °C for 45 s (extension), and 72 °C for 10 min (final extension).

Replicated amplicons were pooled and visualized by electrophoresis in 2.0% agarose gel, and purified using AxyPrep DNA Gel Extraction Kit (Axygen Biosciences, Union City, CA, USA) according to manufacturer’s instructions. Subsequently, the purified amplicons were quantified by Quantus™ Fluorometer (Promega, Madison, WI, USA) and were pooled in equimolar amounts. The DNA library was prepared using the NEXTflex® Rapid DNA-Seq Kit (Bioo Scientific, Austin, TX, USA) and sequenced on the Illumina MiSeq PE300 platform (Illumina, San Diego, CA, USA) at the Shanghai Majorbio Bio-Pharm Technology Co., Ltd., Shanghai, China.

### Operational taxonomic units (OTUs) clustering and taxonomic annotation

The raw sequencing data generated from Illumina MiSeq were pre-processed using Trimmomatic (version 0.39) to remove the known adaptor, specific primers, and low-quality ends (Bolger et al. 2014). We filtered bases with the average quality score below 20 in the tail of reads and set a sliding window of 50 bp. If the average quality score in the window was lower than 20, the back-end base was truncated from the window, the reads below 50 bp after quality control were filtered, and the reads containing ambiguous base were removed. According to overlap relationship between paired-end (PE) reads, paired reads were merged into a sequence with the minimum overlap length of 10 bp using FLASH (version 1.2.7) (Magoč and Salzberg 2011). The overlap region allowed a maximum mismatch ratio of 0.2, and sequences with no matches were discarded.

UPARSE software (version 7.1, http://drive5.com/uparse/) was used to cluster OTUs with 97% similarity cutoff, and chimeras were identified and removed during the clustering process (Costello et al. 2009). The sequence with the highest frequency in each OTU was selected as the representative sequence for further annotation in the process of assigning OTUs. Species classification was annotated for each sequence using ribosomal database project (RDP) classifier (http://rdp.cme.msu.edu/) (Wang et al. 2007), and the comparison threshold was set to 80% in the Silva database (Silva 138/16S) (Li et al. 2017). Based on the taxonomy assignment, all features that refer either to mitochondria, chloroplasts, or archaea were filtered, and the results were aligned to generate the final bacterial OTU table.

### Bioinformatic analysis

The OTUs were annotated for species taxonomy, and the corresponding abundance information of each OTU annotation results in each sample was counted. Before the subsequent analysis, the normalized bacterial OTU table was generated by subsampling randomly based on the minimum number of sample sequences. Community bar charts and Venn charts were used to plot the abundance of each group of FMD and AMD using “stats” package of R software (version 3.3.1, https://www.r-project.org/) (Ji et al. 2017), and the unique bacteria phylum and genus were counted.

Similarities and differences among the microbial communities between FMD and AMD were estimated using cluster heatmap analysis with the R software (packages “pheatmap”) (Perry 2016). Alpha diversity can reflect the diversity of gut microbial composition. At the OTU level, the observed richness (*S*_obs_) index and Shannon index were calculated to measure the diversity of gut microbial composition with Qiime software (http://qiime.org/scripts/assign_taxonomy) (Caporaso et al. 2010). Then the Wilcoxon rank-sum test was used to analyze the significant differences of the alpha diversity index among different groups with the R software (packages “stats”).

Comparative analysis of species diversity in community composition was conducted to explore the similarity or difference of community composition between different groups. Beta diversity analysis between different groups was performed with principle coordinates analysis (PCoA) based on Bray–Curtis distances using the R software (packages “vegan”). Analysis of similarities (ANOSIM), a non-parametric statistical test, was used to test the differences in relative abundance of dominant bacteria and opportunistic pathogens with a two-tailed test with the R software (packages “vegan,” anosim function) (Oksanen et al. 2019). The false discovery rate (FDR) was selected for multiple checks and corrections of *P* value with confidence interval of 0.95.

The metabolic functions and disease-related functions of bacterial communities were predicted using phylogenetic investigation of communities by reconstruction of unobserved states (PICRUSt) software (Langille et al. 2013). Genome sequence data were compared with the Kyoto encyclopedia of genes and genomes (KEGG) database and the nonsupervised orthologous groups (EggNOG) database was used to complete gene functional annotation and classification analysis (Cao et al. 2020). The Wilcoxon rank-sum test was used to analyze the significant difference in functional abundance between different groups.

## Results

### Assessment of sequence data

After strict filtering and quality control of the raw reads, 22,892,300 high-quality clean reads (average 137,905 reads/sample) of FMD and AMD were obtained, generating an average reading length of 375 bp. The rarefaction curves of the *S*_obs_ and Shannon indexes smoothened with increased sequencing quantity (Fig. 1b, c), and Good’s coverage values were higher than 99%. Thus, the sequencing quantity reached saturation, and the data quality was reliable. Therefore, the sequencing data comprehensively reflected the gut microbial information in FMD and AMD under different seasons and ages.

A 97% similarity clustering identified 3,213 effective OTUs of FMD and AMD, and the effective sequences were extracted and screened based on the minimum sample. The OTUs were classified into 20 phyla, 33 classes, 83 orders, 154 families, and 375 genera.

### Gut microbial composition of FMD and AMD

**Fig. 2:**
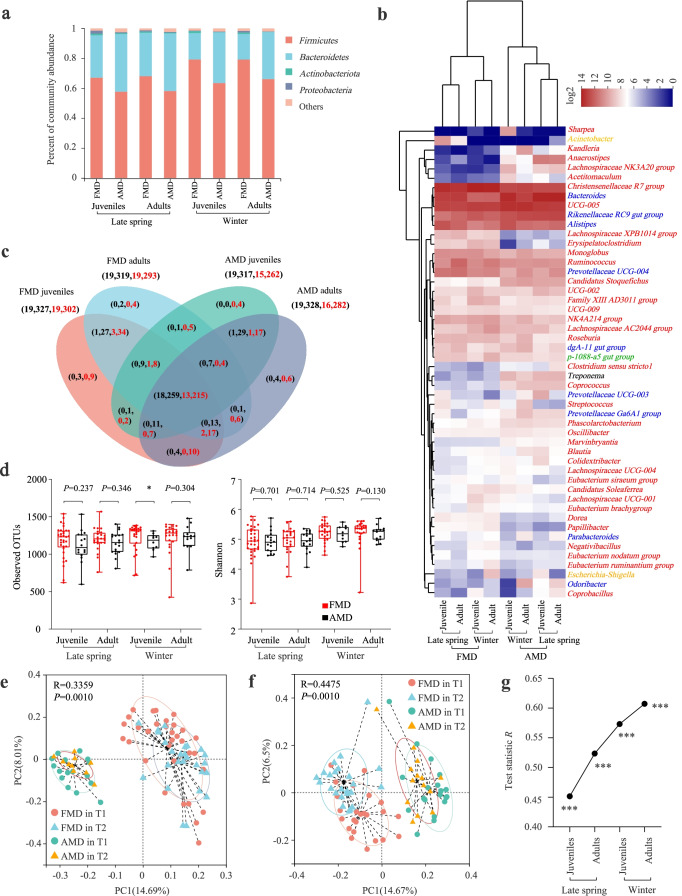
Difference analysis of gut microbiota between FMD and AMD. **a** Histogram of relative abundance of individual bacterial phyla of musk deer. **b** Cluster heatmap analysis based on identifiable bacterial genera with relative abundance of top 50 for musk deer. The red, blue, orange, green, and black letters represented the phyla *Firmicutes*, *Bacteroidetes*, *Proteobacteria*, *Planctomycetes*, and *Spirochaetes*, respectively. **c** Analysis of core and unique bacteria of musk deer at phylum (the left number) and genus (the right number) levels by Venn plots. The black and red numbers represented late spring and winter, respectively. **d** Seasonal variation of α-diversity in gut microbiota of musk deer based on *S*_obs_ and Shannon indexes. PCoA analysis of gut microbial composition between juvenile (**e**) and adult (**f**) FMD and AMD. **g** ANOSIM analysis of gut microbiota between FMD and AMD in the same age and seasons. *P < 0.05 (Wilcoxon rank-sum test), **P < 0.01, and ***P < 0.001. ns, not significant

*Firmicutes* and *Bacteroidetes* were the dominant bacterial phyla across seasons and ages, followed by *Proteobacteria* and *Actinobacteria* in musk deer (Fig. 2a).

The cluster heatmap of the top 50 relative abundances showed that the genera *Bacteroides*, *UCG-005*, *Christensenellaceae R7 group*, *Rikenellaceae RC9 gut group*, *Alistipes*, *Ruminococcus*, *Prevotellaceae UCG 004*, and *Monoglobus* were dominant in both FMD and AMD across seasons and ages (Fig. 2b). However, the genus *NK4A214 group* was dominant in FMD, while *Anaerostipes* and *Candidatus Stoquefichus* were dominant in AMD. The listed dominant genera belong to the phyla *Firmicutes* and *Bacteroides*. Additionally, all the FMD and AMD bacterial genera clustered into one group. In late spring, FMD and AMD shared 18 bacterial phyla and 259 bacterial genera, while in winter, FMD and AMD shared 13 bacterial phyla and 215 bacterial genera (Fig. 2c).

### Difference analysis of gut microbiota between FMD and AMD

The *S*_obs_ and Shannon indexes reflected the richness and diversity of gut microbiota in captive FMD and AMD. The FMD α-diversity was higher than AMD across seasons and ages, but the difference was insignificant (Fig. 2d).

The Bray–Curtis distance algorithm determined the distance between samples, and ANOSIM analysis tested the inter-group and intra-group differences between FMD and AMD.

PCoA showed that all the *R* values were > 0 (*P* = 0.001), indicating significant differences in the gut microbial composition of FMD and AMD in different seasons and ages. The inter-group differences were significantly greater than the intra-group differences (Fig. 2e, f). Adult FMD and AMD had higher β-diversity than juveniles, and the value was higher in winter than in late spring (Fig. 2g).

### Analysis of dominant bacteria differences between FMD and AMD

**Fig. 3:**
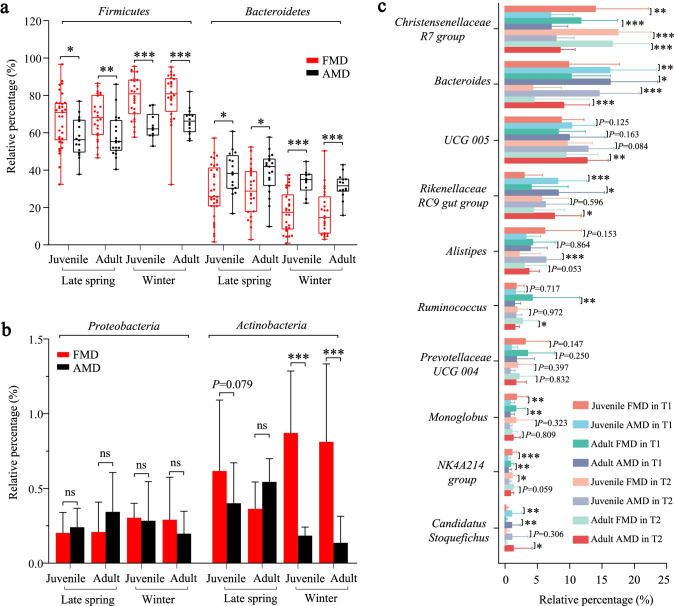
Difference analysis of dominant bacteria between FMD and AMD. **a** Differential analysis of *Firmicutes* and *Bacteroidetes* between FMD and AMD. **b** Differential analysis of *Proteobacteria* and *Actinobacteria* between FMD and AMD. **c** Differential analysis of dominant bacterial genera between FMD and AMD. *P < 0.05 (Wilcoxon rank-sum test), **P < 0.01, and ***P < 0.001. ns, not significant

The Wilcoxon rank-sum test showed that the phyla *Firmicutes* and *Bacteroidetes* differed significantly between FMD and AMD (Fig. 3a). The relative abundance of the phylum *Firmicutes* in FMD was significantly higher than AMD (*P* < 0.05), while the phylum *Bacteroidetes* showed on the contrary a higher relative abundance in AMD than FMD. However, the relative abundance of *Proteobacteria* was lower in FMD than AMD during late spring but higher than AMD in winter, with no significant differences (Fig. 3b). In winter, *Actinobacteria* was significantly higher in FMD than in AMD.

At the genus level, the relative abundances of genera *Christensenellaceae R7 group*, *Ruminococcus*, *Prevotellaceae UCG-004*, *Monoglobus*, and *NK4A214 group* were significantly higher in abundance in FMD than in AMD. In contrast, the genera *Bacteroides*, *UCG-005*, *Rikenellaceae RC9 gut group*, *Alistipes*, and *Candidatus Stoquefichus* were significantly higher in abundance in AMD than in FMD.

### Metabolic function difference analysis

Functional enrichment analysis using the KEGG database identified metabolic function as primary function for gut microbial genes in FMD and AMD. The enriched pathways include carbohydrate metabolism (9.62%), amino acid metabolism (7.19%), energy metabolism (4.27%), metabolism of cofactors and vitamins (4.22%), nucleotide metabolism (2.81%), lipid metabolism (1.80%), glycan biosynthesis and metabolism (1.67%), biosynthesis of other secondary metabolites (1.66%), metabolism of other amino acids (1.18%), and metabolism of terpenoids and polyketides (0.97%).

**Fig. 4:**
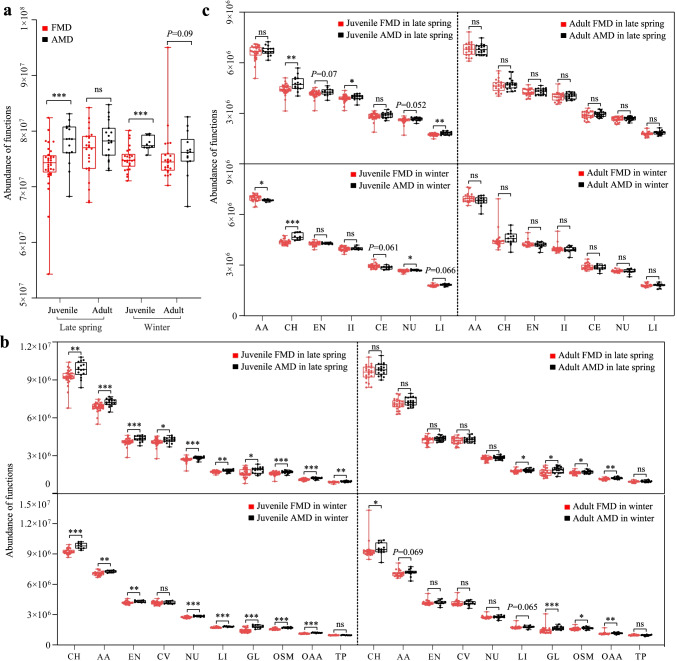
Difference analysis of metabolic function between FMD and AMD based on the KEGG database at level 1 (**a**), at level 2 (**b**), and the EggNOG database (**c**)

At level 1, the metabolic function was significantly enriched in AMD (with stronger enrichment in juvenile than in adult musk deer) than in FMD (Fig. 4a). At level 2, ten metabolic functions were more enriched in AMD than in FMD (Fig. 4b). The difference between juvenile FMD and AMD was higher than between adults.

Additional functional annotation using the EggNOG database also identified metabolic function as the primary function, especially energy production and conversion (6.13%), carbohydrate (6.59%), amino acid (9.85%), nucleotide (3.82%), coenzyme (4.18%), lipid (2.60%), and inorganic ion transport and metabolism (5.73%), respectively. The listed functions were more enriched in AMD (significantly higher in juvenile than adults) than in FMD (Fig. 4c).

**Fig. 5:**
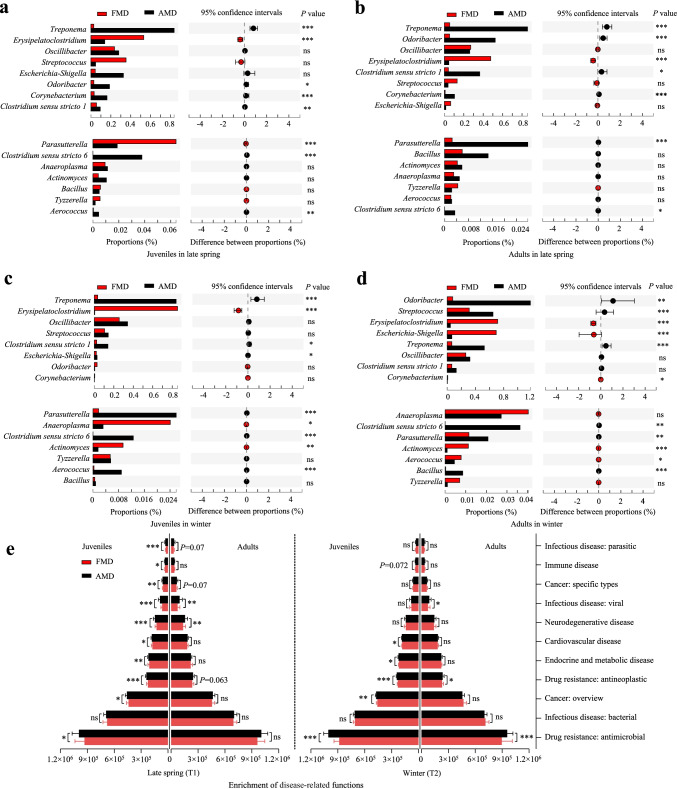
Differences analysis of opportunistic pathogens between FMD and AMD for juveniles in late spring (**a**) and winter (**c**), and for adults in late spring (**b**) and winter (**d**). **e** Differences analysis of disease-related functions between FMD and AMD

The KEGG database showed that gut microbiota with disease-related functions was significantly enriched in AMD than in FMD (Fig. 5e). The difference between juvenile FMD and AMD was higher than between adult musk deer.

### Differences analysis of opportunistic pathogens and disease-related functions

The relative abundance of opportunistic pathogens was less than 0.1% in both FMD and AMD (Fig. 5). In late spring, the relative abundance of the genera *Erysipelatoclostridium* and *Parasutterella* was significantly higher in abundance in juvenile FMD than in juvenile AMD. However, the genera *Treponema*, *Oscillibacter*, *Corynebacterium*, *Clostridium *sensu stricto* 1*, *Clostridium *sensu stricto* 6*, and *Aerococcus* were significantly higher in abundance in juvenile AMD than in juvenile FMD (Fig. 5a). The genus *Erysipelatoclostridium* was significantly higher in abundance adult FMD than in adult AMD. In contrast, the genera *Treponema*, *Odoribacter*, *Clostridium *sensu stricto* 1*, *Corynebacterium*, *Parasutterella*, and *Clostridium *sensu stricto* 6* were significantly higher in abundance in adult AMD than in adult FMD (Fig. 5b).

During winter, the relative abundance of the genera *Erysipelatoclostridium*, *Anaeroplasma*, and *Actinomyces* was significantly higher in juvenile FMD than in juvenile AMD. On the contrary, the genera *Treponema*, *Clostridium *sensu stricto* 1*, *Escherichia-Shigella*, *Parasutterella*, *Clostridium *sensu stricto* 6*, and *Aerococcus* were significantly higher in abundance in juvenile AMD than in juvenile FMD (Fig. 5c). The genera *Erysipelatoclostridium*, *Escherichia-Shigella*, *Corynebacterium*, *Actinomyces*, and *Aerococcus* were significantly higher in abundance in adult FMD than in adult AMD in winter. However, the genera *Odoribacter*, *Streptococcus*, *Treponema*, *Clostridium *sensu stricto* 6*, *Parasutterella*, and *Bacillus* had a significantly higher relative abundance in adult AMD than in adult FMD (Fig. 5d).

## Discussion

The 16S rRNA high-throughput sequencing technology is widely applied to study gut microbial structure and diversity of various endangered wildlife (Antwis et al. 2019; Wei et al. 2019; Guo et al. 2020b). Endangered animals are generally hard to obtain because of their small population size, but their fecal samples are easier to collect without harming animals. Additionally, fecal samples represent the composition and function of microorganisms in the gut microbiota of hosts (Aguirre et al. 2015; Rounge et al. 2018). Collecting fecal samples from endangered wildlife through noninvasive means has become the best research method for conservation biology (Knutie and Gotanda 2018; Ning et al. 2020). Moreover, both artificial breeding and ex situ conservation are effective for maintaining and restoring endangered wildlife populations (Thitaram and Brown 2018; Wang et al. 2016; Willard et al. 1996). Artificial breeding relieves the hunting pressure on wild populations and provides valuable medicinal or raw materials while releasing captive, trained individuals into the wild (Comizzoli and Holt 2019; Silla and Byrne 2019). Thus, this approach is proven and important for conserving rare and endangered species. Varied feeding environments cause diversity and community differences in gut microbiota between captive and wild individuals. For example, gut microbial diversity, metabolic pathways, and cellulose-degrading enzyme activities decreased in captive compared to wild individuals. However, antibiotic resistance genes, heavy metal tolerance genes, the abundance of potential pathogens, and the risk of disease increased in captive compared to wild individuals (Wasimuddin et al. 2017; Chi et al. 2019; Gao et al. 2019). In this study, fecal FMD and AMD samples were obtained by noninvasive means. A 16S rRNA high-throughput sequencing determined the diversity and functional differences of gut microbiota between musk deer. Animal age and sex were controlled, and gut microbiota of captive FMD and AMD was analyzed and compared.

Various factors affect the gut microbiota, including food, the most important source of energy and nutrition for both host and gut microbiota (Wu et al. 2011). The co-evolution of gut microbiota and host, the host genotype (Macke et al. 2017), and genetic polymorphism shape gut microbiota, thus, influencing host susceptibility to disease (Kovacs et al. 2011; De Filippo et al. 2010). Nevertheless, the composition and function of gut microbiota vary in different stages of the host life cycle, increasing in diversity and stability from birth to adult stages while decreasing from adult to old stages (O’Toole and Jeffery 2015). Male and female hosts have different feeding structures, food availability, immunity, metabolite control, and hormone secretion, causing differences in the diversity and function of gut microbiota (Fransen et al. 2017; Johnson et al. 2020). Besides, seasonal variation changes food resources, habitat environment, developmental stage, and migration behavior, possibly all changing host gut microbiota (Baniel et al. 2021; Tong et al. 2020; Shor et al. 2020).

Alpha diversity is the quantitative indicator of host gut microbial diversity, stability, and composition, and is an important evidence of the host health status (Shanahan 2010). Higher alpha diversity indicates a complex and stable gut microbiota, less affected by food variation, and more resistant to external disturbances. Thus, higher alpha diversity is more conducive to host health because hosts can adapt and regulate their homeostasis (Ley et al. 2006; Lang et al. 2018). This study showed that captive FMD had a higher alpha diversity than captive AMD of the same age and season, consistent with previous studies (Hu et al. 2017). FMD and AMD are different musk deer species. Hence, the gut microbial diversity from this study is inadequate for comparing the health status of both species under a captive environment. However, previous studies showed that FMD has a larger suitable habitat than AMD (Jiang et al. 2020). FMD is the most widely distributed deer species with the highest population in China, followed by AMD (National Forestry and Grassland Administration 2009). Musk deer breeding in captivity has taken over 60 years since the 1960s, but different studies hypothesize a higher alpha diversity in wild FMD than in wild AMD (Li et al. 2017). Therefore, higher alpha diversity of gut microbiota probably benefits FMD adaption to different habitats.

Gut microbiota is rich in genes for the metabolism of carbohydrates, amino acids, fats, cellulose, short-chain fatty acids (SCFAs), bile acids, and the synthesis of methane and vitamins (Sasaki et al. 2019). These genes synthesize enzymes and regulate biochemical metabolic pathways necessary for metabolizing various substances for host sustenance (Tremaroli and Backhed 2012). Host metabolism is closely related to the growth and development of the host. As hosts age, the gut microecosystem gradually forms complex digestive and metabolic functions. The diversity and stability of the gut microecosystem ensure normal metabolic function, nutrient digestion, and absorption by the host. However, imbalanced microbiota significantly alters the host metabolic and physiological processes, causing host susceptibility to diseases, especially metabolic disease (Torres-Fuentes et al. 2017).

Dominant bacteria are key in metabolic function. This study showed that the phyla *Firmicutes* and Bacteroidetes were dominant, accounting for > 90% diversity in musk deer species across different seasons and ages. The above results are consistent with other results on the gut microbial composition in FMD, AMD, and other ruminants (Hu et al. 2017; Zhao et al. 2019a, b; Fountain-Jones et al. 2020). In ruminants, the phylum *Firmicutes* promotes fiber and cellulose degradation into volatile fatty acids, thus, facilitating food digestion, animal growth, and development (Wang et al. 2018). In this study, the relative abundance of *Firmicutes* was significantly higher in FMD than in AMD, while *Bacteroidetes* was significantly higher in abundance in FMD than in AMD. The genera *Bacteroides*, *UCG-005*, *Christensenellaceae R7 group*, *Rikenellaceae RC9 gut group*, *Alistipes*, *Ruminococcus*, *Prevotellaceae UCG 004*, and *Monoglobus* were dominant in both FMD and AMD. Among these, the genera *Christensenellaceae R7 group*, *Ruminococcus*, and *Prevotellaceae UCG 004* belong to *Firmicutes*. Studies have shown that the genus *Ruminococcus* produces numerous cellulase and hemicellulase enzymes through fermentation in the rumen of ruminants (Matulova et al. 2008; La Reau et al. 2016). These enzymes convert the dietary fiber in food into various nutrients needed by the host, and play a key role in food digestion and carbohydrate metabolism (La Reau and Suen 2018). The genus *Christensenellaceae R7 group*, also abundant in the rumen of ruminants, is very important to the structure and function of the host intestinal tract and is mainly involved in amino acid, peptide, and lipid metabolism of the host (Waters and Ley 2019). The genus *Prevotellaceae UCG 004* degrades polysaccharides and produces SCFAs (Heinritz et al. 2016). Moreover, the genera *Bacteroides*, *Alistipes*, and *Rikenellaceae RC9 gut group* belong to *Bacteroidetes*. The genus *Bacteroides* improves ruminant metabolism by metabolizing bile acid, protein, and fat, and regulating carbohydrate metabolism. In comparison, the genus *Alistipe* metabolizes short-chain fatty acids. Both *Bacteroides* and *Alistipe* contain bile-tolerant microorganisms (David et al. 2014) and increase lipid metabolism by regulating the production of acetic acid (Yin et al. 2018). However, the genus *Rikenellaceae RC9 gut group* also metabolizes lipids (Zhou et al. 2018). Since both musk deer are typical ruminants, the identified dominant bacteria are critical for food digestion, nutrient absorption, and energy metabolism.

The relative abundance of genera *Christensenellaceae R7 group*, *Ruminococcus*, *Prevotellaceae UCG-004*, and *Monoglobus* were significantly higher in FMD than in AMD. In contrast, genera *Bacteroides*, *UCG-005*, *Rikenellaceae RC9 gut group*, and *Alistipes* were more dominant in AMD than in FMD. Moreover, gene function annotation and prediction showed that the expression of metabolic function was higher in AMD gut microbiota than in FMD. FMD is the smallest animal in the *Moschidae* family, and the body size of AMD is significantly larger than that of FMD (Wu and Wang 2006). Consequently, AMD requires higher energy and substance metabolism for growth, development, and activity than FMD. Moreover, higher gut microbial metabolism is conducive for maintaining higher energy and substance metabolism in AMD.

Furthermore, the relative abundance of opportunistic pathogens and the expression of disease-related functions were significantly higher in AMD than in FMD, suggesting that captive AMD may be at greater risk of intestinal diseases than FMD. For example, the genus *Treponema* is associated with dysentery, which causes severe colon inflammation. Moreover, gout patients showed an increased relative abundance of genus *Erysipelatoclostridium* in their intestinal tract (Shao et al. 2017). The genus *Odoribacter* may cause several intestinal diseases, such as colitis, necrotizing enteritis, and gastroenteritis (Meng et al. 2019). Most species in *Corynebacterium* are opportunistic pathogens that cause endocarditis, bacteremia, and respiratory tract, urinary tract, and various other types (Aravena-Roman et al. 2012). Besides, the genus *Parasutterella* can cause chronic inflammation (Peng et al. 2019). Altogether, the above potential pathogens may explain the high mortality of captive AMD.

In conclusion, this study systematically and comprehensively analyzed the differences in gut microbial structure and function between FMD and AMD using 16S rRNA gene analysis of fecal samples from 166 captive musk deer in different seasons and ages. The results showed that the alpha diversity was higher in FMD than in AMD. There were significant differences in the gut microbial composition between both musk deer. The ß-diversity between adult FMD and AMD was higher than between juvenile individuals, and the value was higher in winter than in late spring. The species differences indicate that the relative abundance of the phylum *Firmicutes* and the genera *Christensenellaceae R7 group*, *Ruminococcus*, *Prevotellaceae UCG-004*, and *Monoglobus* were significantly higher in FMD than in AMD. In contrast, the phylum *Bacteroidetes* and the genera *Bacteroides*, *UCG-005*, *Rikenellaceae RC9 gut group*, and *Alistipes* were significantly higher in abundance in AMD than in FMD. The relative abundance of most opportunistic pathogens in AMD was significantly higher than in FMD. Additionally, the metabolic functions and disease-related functions of gut microbiota were significantly higher in AMD than in FMD. The combined metagenome and metabolomic results from this study are important for evaluating the artificial breeding environment and future reintroduction programs.

## Data Availability

The datasets generated for this study can be found in the NCBI Sequence Read Archive under BioProject PRJNA725631 with the accession number SUB9547782 (https://dataview.ncbi.nlm.nih.gov/object/PRJNA725631?reviewer=e2rcdic27nir1a7qhp8unlk9s6).
